# LncRNA GTF3C1 promotes diabetic corneal wound healing by regulating GABARAP and PTEN to augment autophagy

**DOI:** 10.1186/s40662-025-00448-y

**Published:** 2025-08-11

**Authors:** Danling Liao, Wenqu Chen, Yuyang Deng, Shijia Wei, Li Wang, Jianzhang Hu

**Affiliations:** 1 Department of Ophthalmology, Fujian Medical University Union Hospital, 29 Xinquan Road, Fuzhou, 350005 China; 2 Department of Experimental and Translational Ophthalmology, University Medical Center, Johannes Gutenberg University, 55131 Mainz, Germany

**Keywords:** Autophagy, Diabetic corneal neuropathy, GABARAP, LncRNA GTF3C1, Nerve regeneration, PTEN

## Abstract

**Background:**

Diabetic keratopathy (DK) is a common ocular complication of diabetes, with its progression closely linked to autophagy regulation. This study aims to explore the role of long non-coding RNAs (lncRNAs) in modulating autophagy during diabetic pathogenesis, focusing on lncRNA general transcription factor IIIC subunit 1 (GTF3C1) and its potential as a therapeutic target for diabetic corneal neuropathy (DCN).

**Methods:**

High-throughput sequencing identified dysregulated lncRNAs in the trigeminal ganglia of diabetic mice. Functional validation included mechanistic studies on lncRNA GTF3C1, miR-542-3p, and autophagy-related targets. Autophagy activity, corneal nerve density, and epithelial healing were quantified using quantitative real-time polymerase chain reaction (qRT-PCR), immunofluorescence, and histology in diabetic models.

**Results:**

lncRNA GTF3C1 was significantly downregulated in diabetic trigeminal ganglion (TG). It functioned as a molecular sponge for miR-542-3p, alleviating its repression on GABA type A receptor-associated protein (GABARAP) and phosphatase and tensin homolog (PTEN), thereby enhancing autophagy activity. This process promoted corneal nerve fiber regeneration and epithelial wound healing in diabetic mice.

**Conclusions:**

Our findings highlight lncRNA GTF3C1 as a critical regulator of autophagy in diabetic corneal nerves, offering a potential diagnostic and therapeutic target for DCN. This study provides molecular insights into the pathogenesis of DCN and lays the groundwork for future clinical strategies.

**Supplementary Information:**

The online version contains supplementary material available at 10.1186/s40662-025-00448-y.

## Highlights

- Downregulated lncRNA GTF3C1 in diabetic trigeminal ganglia is linked to impaired autophagy and diabetic keratopathy progression.
- lncRNA GTF3C1 cushions miR-542-3p’s effect to enhance autophagy via GABARAP/PTEN, promoting corneal nerve regeneration and epithelial repair.
- lncRNA GTF3C1 serves as a therapeutic/diagnostic target for diabetic corneal neuropathy by regulating autophagy and clinical strategy development.

## Background

Diabetes is a common chronic metabolic disease. Approximately half of diabetic patients develop diabetic keratopathy (DK), which threatens the visual quality of diabetic patients [1, 2]. The characteristic pathological markers of DK include decreased corneal sensitivity, superficial punctate keratopathy, delayed epithelial wound healing, and persistent corneal ulcers [3]. The corneal nerve primarily originates from the ophthalmic division of the trigeminal nerve, which innervates the cornea, secretes neuropeptides to nourish adjacent epithelial and immune cells, and exerts crucial functions in corneal wound healing [1, 4, 5]. Currently, corneal nerve sensory and nutritional dysfunction caused by hyperglycemia is considered a key cause of DK [1, 6, 7]. However, the pathogenesis of diabetic corneal neuropathy (DCN) is still not fully understood, and understanding the underlying mechanisms and identifying innovative therapeutic interventions remains a critical research imperative in addressing this condition.

There is growing evidence that functional defects in autophagy serve as important pathogenic mechanisms of diabetes and its complications [8, 9]. Autophagy is the process of delivering intracellular substances to lysosomes for degradation, and it is critical for cell homeostasis and the body’s adaptation to environmental stress [8, 10]. Light chain 3 beta (LC3B), a core autophagosome regulator, undergoes lipidation (conversion to LC3-II) that positively correlates with autophagosome quantity, serving as a canonical autophagic activity biomarker [11]. Conversely, selective autophagy adaptor sequestosome-1 (P62) is degraded via autophagic flux, exhibiting an inverse correlation with autophagic activation [12]. Studies in recent years have suggested that autophagy dysfunction occurs in diabetic corneal nerves and that rapamycin accelerates diabetic corneal healing by activating autophagy [13]. In addition, the upregulation of the expression of autophagy related gene 5 (ATG5), ATG4B, and ATG4D in the trigeminal nerve has been shown to promote the regeneration of diabetic corneal nerves and promote the healing of corneal epithelial wounds [13–15]. Therefore, autophagy impairment has been confirmed to be a key link in DCN, and moderate autophagy activation may become a novel treatment direction for diabetic corneal wound healing.

Long non-coding RNAs (lncRNAs) are non-coding RNAs > 200 nt in length that can interact with proteins and RNA in a variety of functional forms and regulate gene expression at various levels, including the epigenetic level, transcription level, and posttranscription level [16]. Compared with protein-coding genes, lncRNAs have lower sequence conservation. This characteristic enables lncRNAs to exert specialized functions in disease-specific regulation [17]. lncRNAs in the cytoplasm often serve as “molecular decoys” to competitively bind to microRNAs (miRNAs), thereby blocking the silencing effect of miRNAs on target genes [18]. Some studies have confirmed that lncRNAs act as autophagy regulatory molecules in diabetes by competitively binding to miRNAs. LncRNA diabetic cardiomyopathy-related factor (DCRF), affects myocardial autophagy through miR-551b-5p to improve cardiac function in diabetic rats [19]. In addition, lncRNA XIST attenuates diabetic peripheral neuropathy by inducing autophagy through the miRNA-30d-5p/SIRT1 axis [20]. However, the prospect of lncRNAs in DCN and the molecular mechanisms involved in the autophagy regulatory network are still unknown. Deciphering the lncRNA profile in DCN and elucidating their pathogenic mechanisms advance precision molecular diagnostics and targeted therapeutic.

In this study, high-throughput lncRNA sequencing revealed general transcription factor IIIC subunit 1 (GTF3C1) as a novel autophagy-regulating lncRNA in DCN. We further elucidated its mechanistic role in modulating autophagic pathways, identifying its potential as both a diagnostic biomarker and therapeutic target for DCN degeneration.

## Materials and methods

### Animals

Male C57BL/6 J mice aged 6–8 weeks were obtained from Beijing SPF Biotechnology Co., Ltd. (Beijing, China). All procedures involving laboratory animals were conducted in compliance with the guidelines approved by the Institutional Animal Care and Use Committee of Fujian Medical University (IACUC FJMU 2021–0454) and adhered to the ophthalmology statement regarding the use of animals in ophthalmic and vision research. To induce type 1 diabetes, mice were administered streptozotocin (STZ; Sigma‒Aldrich, USA) via intraperitoneal injection. The experimental group received low-dose STZ (50 mg/kg) intraperitoneally over five consecutive days, while the control group was injected with citrate-citric acid buffer. Both groups were maintained on standard diets. Fasting blood glucose levels were assessed prior to the injections and subsequently every 4 weeks for up to 16 weeks. Mice exhibiting blood glucose levels exceeding 16.7 mmol/L were identified as diabetic.

### Assessment of corneal sensitivity

Corneal sensitivity was assessed with a Cochet-Bonnet esthesiometer (Luneau Ophthalmology, France). A blinking response was observed in unanesthetized mice when a nylon filament, deflected approximately 5° and applied perpendicularly to the cornea, triggered a positive blink reflex. The filament length was decreased by 5 mm increments until a positive reaction occurred. Measurements were conducted on both eyes of each mouse, with six repetitions performed following corneal epithelial degradation on the fifth day.

### Evaluation of corneal epithelial wound healing

Corneal epithelial wound healing in mice was assessed following anesthesia induced by intraperitoneal injection of 1.25% tribromoethanol, with additional local ocular surface anesthesia provided by topical 0.5% promethazine hydrochloride. A 2.5-mm diameter area of the corneal epithelium was excised using an AlgerBrush II rotary tool (Alger Inc., USA), followed by the application of ofloxacin ophthalmic ointment to prevent infection. The epithelial wound area was visualized by staining with 0.25% sodium fluorescein and subsequently examined at 0, 12, 24, and 36 h post-injury under a microscope equipped with cobalt blue illumination. ImageJ software was utilized for quantitative analysis to calculate the percentage of epithelial defect area in fluorescein-stained corneal regions. Each experimental group consisted of six mice, and independent replicate experiments were conducted to ensure reproducibility.

### Whole-mount staining of corneal nerves

Five days post corneal epithelial wounding, intact corneas were dissected and fixed in Zamboni's solution for 2 h at 4 ℃. Following permeabilization with 0.3% Triton X-100 and blocking in 5% normal goat serum, tissues were incubated overnight at 4 ℃ with anti-III-tubulin antibody (657,404, Biolegend, USA). Confocal z-stack imaging was performed using a Leica microscope (20 × objective, z-step 5 μm). Nerve fiber density was quantified by calculating β III-tubulin^+^ area percentage (vs. total corneal area) through threshold analysis in ImageJ. Each experimental group included six mice for independent replicates.

### Administration of subconjunctival injections

To modulate the expression of target RNA in experimental mice, subconjunctival injections were performed. Following the induction of general and topical ocular anesthesia, 5 µL of the respective solutions were administered into the bulbar conjunctiva using a microsyringe. Injections were performed at multiple sites within the bulbar conjunctiva. The timing of administration included 24 h prior to injury, immediately at the time of injury, and 24 h post-injury. Supplementary Table S1 shows the injection solutions. Each experimental group comprised six mice, and independent replicate experiments were conducted to ensure reproducibility.

### Western blot analysis

Trigeminal ganglion (TG) tissues were homogenized in RIPA Buffer, and the proteins extracted were separated using 10%–15% sodium dodecyl sulfate polyacrylamide gel electrophoresis (SDS-PAGE) before being transferred to polyvinylidene fluoride (PVDF) membranes. The membranes were incubated with the appropriate primary and secondary antibodies. Protein bands were detected using an enhanced chemiluminescent substrate. The primary antibodies employed are shown in Supplementary Table S2. Each experiment was conducted with three independent replicates.

### Immunofluorescence

TG tissues were harvested, embedded, and stored at − 80 °C. Sections of 5 μm thickness were obtained for fixation. To minimize nonspecific interactions, tissue samples were permeated with 0.3% Triton X-100 and subsequently treated with 10% goat serum. The sections were incubated overnight with primary antibodies and subsequently with Alexa Fluor-conjugated secondary antibodies. Fluorescence imaging utilized a Leica fluorescence microscope (Germany). The primary antibodies used are shown in Supplementary Table S2. Each experiment was conducted with three independent replicates.

### Transmission electron microscopy

Freshly dissected TG tissues were promptly sectioned into 1 mm^3^ cubes and fixed using 1% osmium tetroxide. The fixed samples were sequentially dehydrated through a graded series of ethanol and acetone, with each step taking 15 min. The tissues were then embedded in epoxy resin and polymerized at 60 ℃. Ultrathin Sects. (80 µm) were stained with 2% uranyl acetate and lead citrate. Autophagosome quantification and morphological analysis were performed using a Hitachi HT-7700d transmission electron microscope (Tokyo, Japan). Each group contained three independent samples.

### lncRNA and miRNA sequencing

RNA sequencing was conducted using fresh TG tissues from diabetic mice and control mice (n = 3 per group). High-throughput RNA sequencing was performed by Kangcheng Biotech, Inc. (Shanghai, China). The edgeR analytical tool was used to identify differentially expressed RNAs in paired samples with counts per million reads greater than 1 in each group. RNAs that were differentially expressed met the criteria of *P* < 0.05 and |log2 fold change| value ≥ 0.585.

### Quantitative real-time polymerase chain reaction (qRT-PCR)

Using TRIzol (Invitrogen, USA) total RNA were extracted from freshly collected TG tissues. Subcellular localization of lncRNAs was analyzed by separating cytoplasmic and nuclear RNA fractions with a PARIS Kit (Ambion, Life Technologies, USA). Gene expression levels were quantified using an ABI 7500 Real-Time PCR system (Life Technologies, Singapore) following the conversion to Complementary DNA (cDNA). Primers were custom-designed and synthesized by Sangon Biotech in Shanghai, China. The primer sequences are detailed in Supplementary Table S3. Each experimental group included at least three mice, and independent replicate experiments were conducted to ensure reproducibility.

### Dual-luciferase reporter gene assay

Potential binding sites for miR-542-3p on GABA type A receptor-associated protein (GABARAP) and phosphatase and tensin homolog (PTEN) were predicted using the TargetScan algorithm, while interactions between lncRNA GTF3C1 and miR-542-3p were identified through manual nucleotide sequence analysis. Plasmid vectors encoding either wild-type or mutant versions of lncRNA GTF3C1, GABARAP and PTEN were synthesized (Hanbio, Shanghai, China). Mutations were introduced at both predicted binding sites within the lncRNA GTF3C1 sequence. Cells were co-transfected with either miR-542-3p or a negative control and wild-type or mutant plasmids, followed by luciferase activity measurement 48 h later. Each experimental condition included three mice, and independent replicate experiments were conducted to ensure reliability.

### Statistical analyses

At least three independent experiments were conducted to ensure robust results. GraphPad Prism (v.9.5.1, San Diego, USA) was utilized for data analysis. Results are presented as means ± standard errors of the means (SEMs). Group comparisons were conducted using either an unpaired two-tailed Student’s t-test or one-way ANOVA, based on the experimental design. Statistical significance was defined as a *P* value < 0.05, with specific thresholds denoted as follows: ns, not significant (*P* > 0.05); **P < 0.01; ***P < 0.001; and ****P < 0.0001.

## Results

### DElncRNA expression profiles and characteristics of the trigeminal ganglion tissue in diabetic mice

**Fig. 1:**
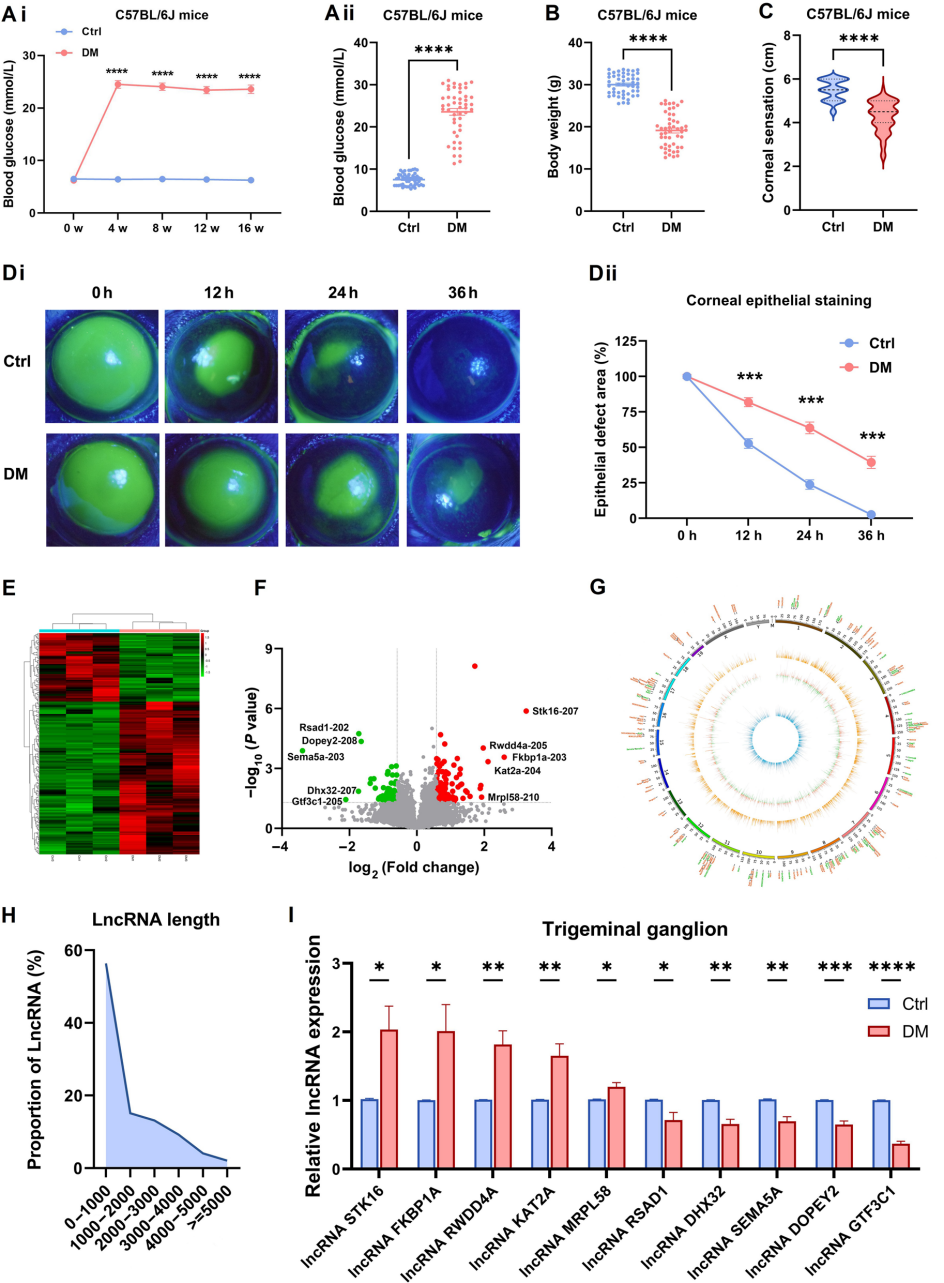
Differentially expressed long non-coding RNAs (DElncRNA) expression profiles and characteristics of the trigeminal ganglion (TG) tissue in diabetic mice. **A**: Blood glucose values of control (Ctrl) and diabetic (DM) mice. **Ai**: Blood glucose at 0, 4, 8, 16 weeks after intraperitoneal injection (n = 50 per group). **Aii**: The differences in blood glucose levels between the two groups at 16 weeks (n = 50 per group). **B**: Body weight of control (Ctrl) and diabetic (DM) mice at 16 weeks (n = 50). **C**: Corneal sensitivity of Ctrl and DM mice at 16 weeks (n = 50 per group). **D**: Corneas stained with fluorescein sodium at 0, 12, 24, and 36 h in Ctrl and DM mice after debridement (n = 6 per group). **Di**: The fluorescent staining of corneal epithelium. **Dii**: The percentage of epithelial defect area. **E**: Heatmap of the DElncRNAs in three pairs of TG tissues from Ctrl and DM mice (n = 3 per group). **F**: Volcano plot of DElncRNAs. Red, upregulated; Green, downregulated. **G**: Circos plots characterizing DElncRNAs. Outer: DElncRNAs; Orange circle: average lncRNA expression in diabetic TG; Blue: average lncRNA expression in control TG; Circle between orange and blue: the values represent the log (fold change) of diabetic TG; Orange: upregulated; Green: downregulated. **H**: DElncRNAs length distribution. **I**: Quantitative real-time polymerase chain reaction (qRT-PCR) validated the differential expression of lncRNAs in TG tissue between Ctrl and DM mice (n = 8 per group). *P < 0.05; **P < 0.01; ***P < 0.001; ****P < 0.0001

In this study, we induced type 1 diabetes in mice via the intraperitoneal injection of STZ. After induction, compared with age-matched controls, STZ-injected mice exhibited a persistent hyperglycemic state (Fig. 1A) and lost a significant amount of body weight (Fig. 1B); moreover, corneal sensitivity was significantly reduced (Fig. 1C), and corneal epithelial wound healing was significantly delayed (Fig. 1D), confirming that diabetic mice develop corneal neuropathy and delays in corneal epithelial wound healing.

Corneal nerve fibers are derived mainly from the TG tissue. To understand the expression profiles of lncRNAs in diabetic corneal nerves, we collected TG tissue from diabetic and control mice for RNA high-throughput sequencing analysis. A total of 194 differentially expressed lncRNAs (DElncRNA) were identified between TG tissue from diabetic (DM) mice and TG tissue from control mice (*P* < 0.05, fold change > 1.5), with 134 upregulated and 60 downregulated lncRNAs (Fig. 1E–F). A Circos plot was generated to visualize the expression level and chromosomal location of each lncRNA (Fig. 1G). The lengths of the lncRNA transcripts were mostly within 2000 nt (Fig. 1H). On the basis of |log2 fold change, we selected five significantly upregulated lncRNAs and five downregulated lncRNAs to verify their differential expression via qRT-PCR. The results were consistent with the sequencing trend. The most significant difference was observed for the lncRNA GTF3C1 (Fig. 1I). On the basis of the fold difference and expression abundance, we chose the lncRNA GTF3C1 for further investigation.

### Identification of the lncRNA GTF3C1 as an autophagy activator in diabetic TG tissue

**Fig. 2:**
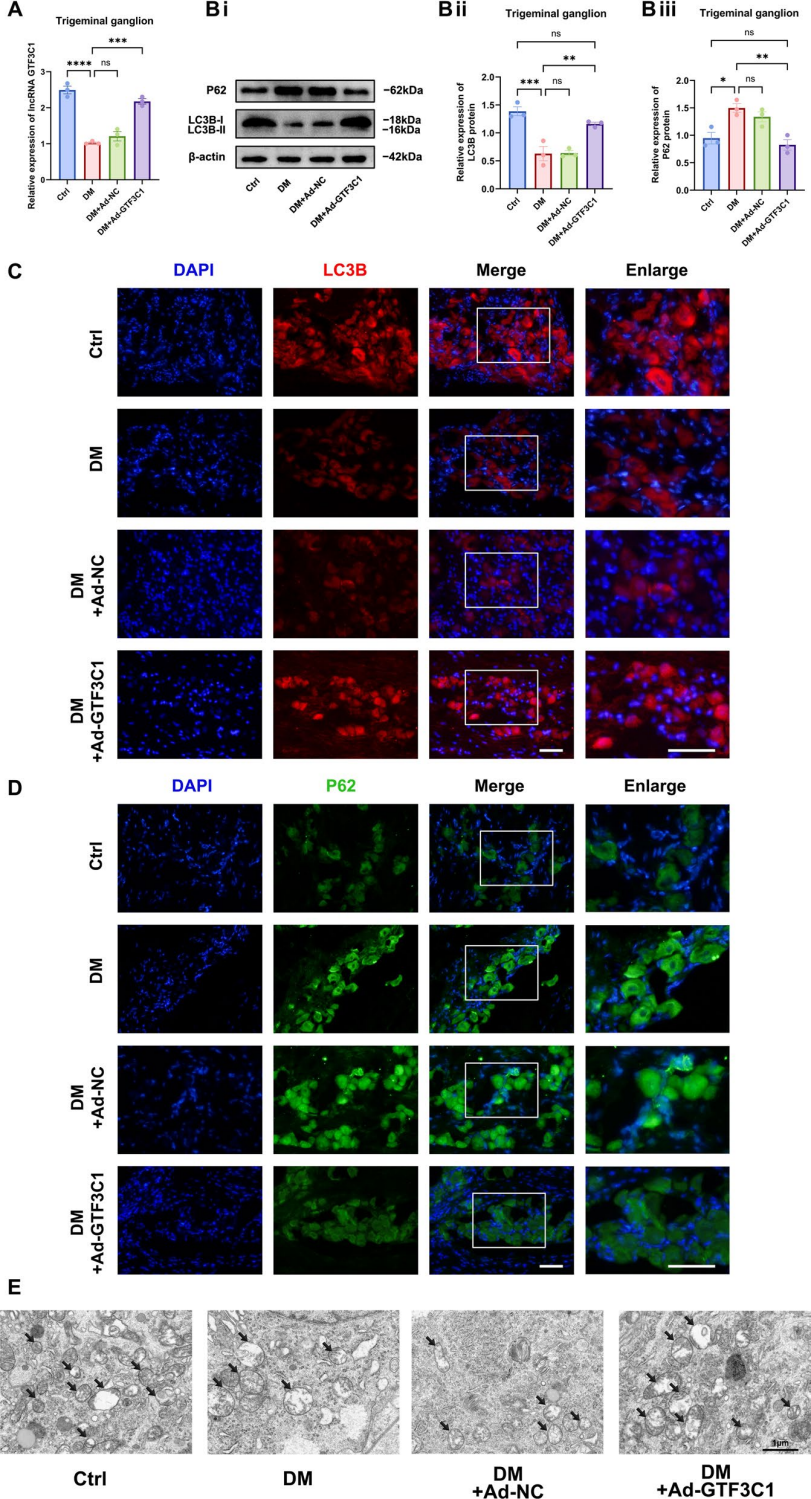
Identification of the lncRNA GTF3C1 as an autophagy activator in diabetic TG tissue. **A**: Quantitative real-time polymerase chain reaction (qRT-PCR) validated the differential expression of lncRNA GTF3C1 in trigeminal ganglion (TG) tissue of control mice (Ctrl), diabetic mice (DM), adenovirus negative control treated diabetic mice (DM + Ad-NC), and Ad-GTF3C1 treated diabetic mice (DM + Ad-GTF3C1) (n = 3 per group) on day 5 after subconjunctival injection. **B**: Western blot analysis clarified autophagy proteins expression in TG tissue of Ctrl, DM, DM + Ad-NC and DM + Ad-GTF3C1 (n = 3 per group). **Bi**: Western blot bands of LC3B and P62 proteins. Quantified intensities of Western blot bands for LC3B (**Bii**) and P62 (**Biii**) compared with β-actin. **C**: Immunofluorescence analysis showed LC3B protein expression in TG tissue of each group (n = 3 per group). **D**: Immunofluorescence analysis showed P62 protein expression in TG tissue of each group (n = 3 per group). **E**: Autophagosomes (black arrows) in TG tissue of each group under a transmission electron microscope (n = 3 per group). Scale bar (including enlarged image): 50 μm. ns,not significant; *P < 0.05; **P < 0.01; ***P < 0.001; ****P < 0.0001

Autophagy impairment is an important factor that delays corneal damage repair in diabetes. To study whether the lncRNA GTF3C1 is involved in the regulation of autophagy in the TG tissue, we constructed the lncRNA GTF3C1-overexpressing adenovirus Ad-GTF3C1. The subconjunctival injection of Ad-GTF3C1 successfully upregulated the expression of the lncRNA GTF3C1 in diabetic TG tissue (Fig. 2A). The experimental results revealed that LC3B protein levels were significantly reduced, while P62 protein expression was elevated in the TG tissue of DM vs. control mice, indicating that autophagy was inhibited as expected (Fig. 2B–D). Overexpression of lncRNA GTF3C1 markedly upregulated LC3B protein levels and downregulated P62 protein expression in diabetic TG tissue, suggesting that the lncRNA GTF3C1 increased the level of autophagy in diabetic TG tissue (Fig. 2B–D). In addition, transmission electron microscopy (TEM) revealed that the overexpression of the lncRNA GTF3C1 led to a higher accumulation of autophagosomes in diabetic TG tissue (Fig. 2E). These results suggest that the lncRNA GTF3C1 had an autophagy agonist function in diabetic TG tissue.

### The lncRNA GTF3C1 promotes diabetic corneal epithelial repair and nerve regeneration by enhancing autophagy

**Fig. 3:**
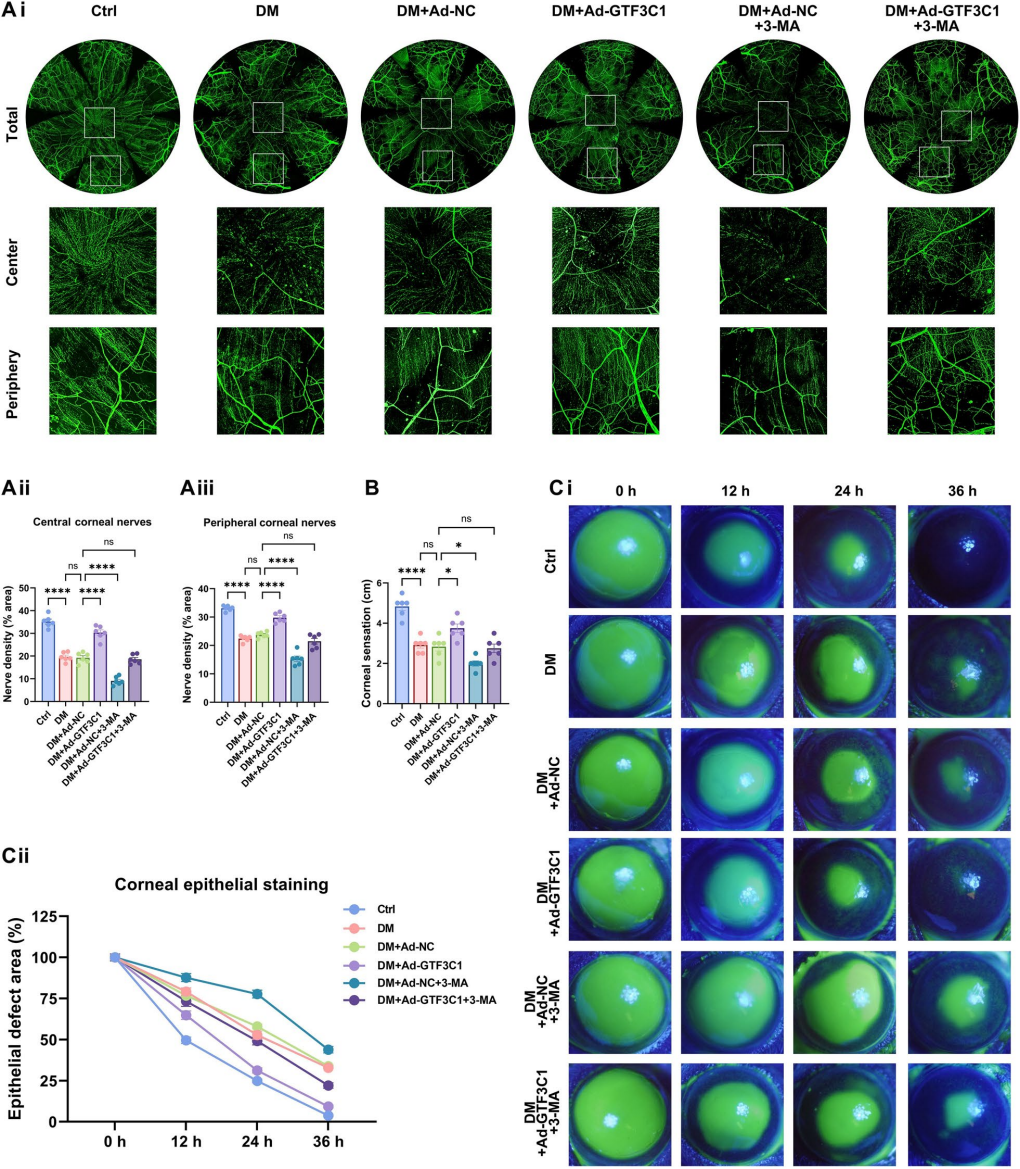
The lncRNA GTF3C1 promotes diabetic corneal epithelial repair and nerve regeneration by enhancing autophagy. **A**: Corneal whole-mount staining of control mice (Ctrl), diabetic mice (DM), Ad-NC treated diabetic mice (DM + Ad-NC), Ad-GTF3C1 treated diabetic mice (DM + Ad-GTF3C1), Ad-NC and 3-methyladenine (3-MA) treated diabetic mice (DM + Ad-NC + 3-MA), Ad-GTF3C1 and 3-MA treated diabetic mice (DM + Ad-GTF3C1 + 3-MA) on day 5 after debridement (n = 6 per group). **Ai**: The fluorescent images of corneal nerve staining. **Aii**: The central corneal nerve density. **Aiii**: The peripheral corneal nerve density. **B**: Corneal sensation in each group on day 5 after debridement (n = 6 per group). **C**: Corneas stained with fluorescein sodium of each group at 0, 12, 24, and 36 h after debridement (n = 6 per group). **Ci**: Fluorescent staining of the corneal epithelium. **Cii**: The percentage of epithelial defect area. ns, not significant; *P < 0.05; ****P < 0.0001

To assess the effect of the lncRNA GTF3C1 in the repair of diabetic corneal injury, we performed subconjunctival injection of adenovirus-negative control (Ad-NC) and adenovirus containing the GTF3C1 gene (Ad-GTF3C1) in diabetic mice. Overexpression of the lncRNA GTF3C1 markedly increased corneal nerve fiber density and enhanced corneal sensitivity 5 days following corneal epithelial scraping, relative to untreated conditions (Fig. 3A, B). Moreover, the area of the corneal epithelial defect decreased significantly at 12, 24, and 26 h after scraping, suggesting that the overexpression of the lncRNA GTF3C1 promoted the healing of corneal epithelial tissues (Fig. 3C). In addition, to determine whether the lncRNA GTF3C1 affected diabetic cornea wound healing by regulating autophagy, we injected Ad-GTF3C1 into the subconjunctiva of diabetic mice concurrently with the autophagy inhibitor 3-methyladenine (3-MA). The results demonstrated that 3-MA counteracted the beneficial effects of Ad-GTF3C1 on diabetic corneal epithelial and nerve restoration (Fig. 3A–C). These results indicate that the overexpression of the lncRNA GTF3C1 promoted epithelial healing and nerve restoration in the corneas of diabetic mice by enhancing autophagy.

### The lncRNA GTF3C1 targets miR-542-3p to regulate autophagy in diabetic TG tissue

**Fig. 4:**
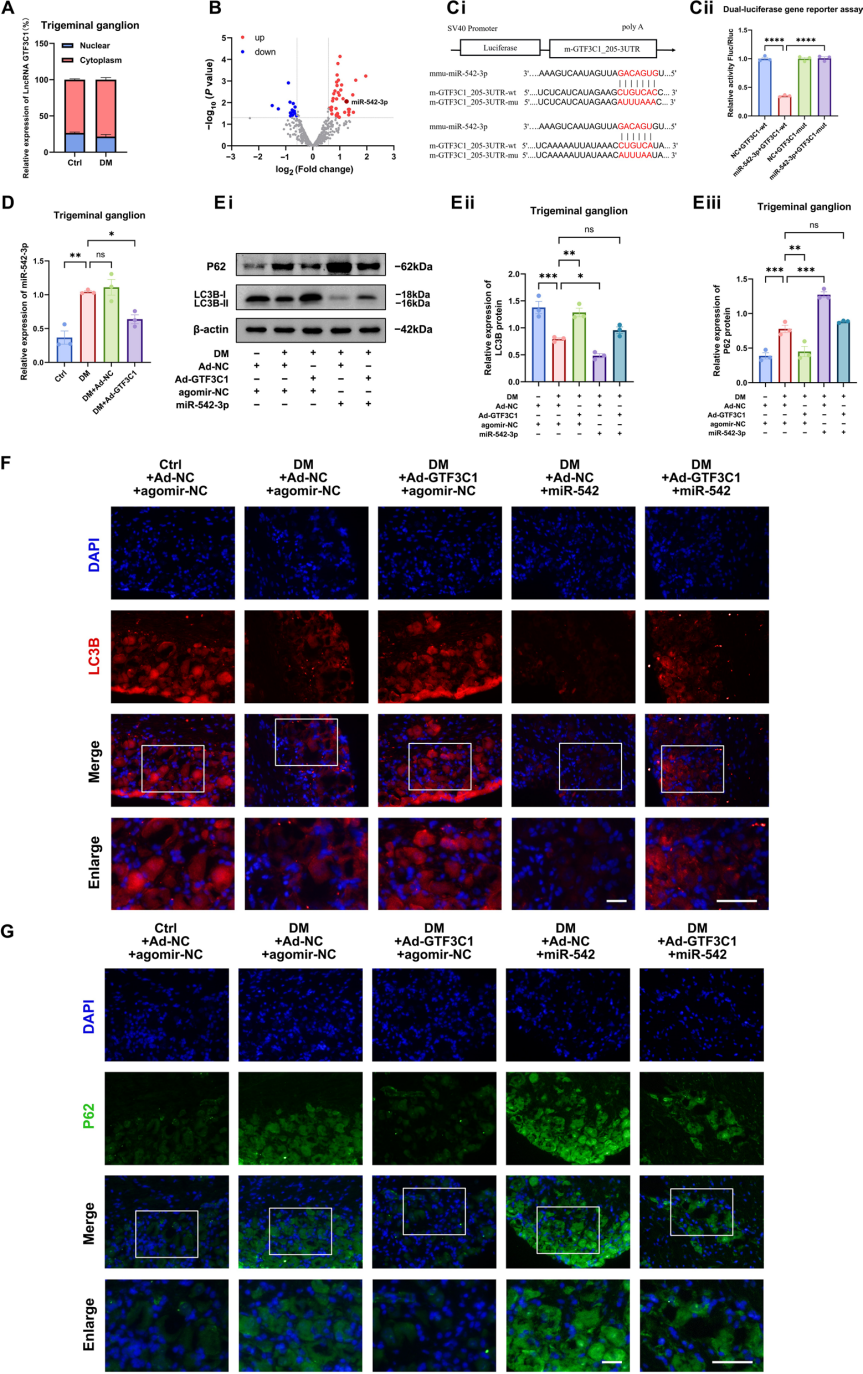
The lncRNA GTF3C1 targets miR-542-3p to regulate autophagy in diabetic trigeminal ganglion (TG) tissue. **A**: Relative expression of lncRNA GTF3C1 in the nucleus and cytoplasm of TG tissues from control (Ctrl) and diabetic (DM) mice detected by quantitative real-time polymerase chain reaction (qRT-PCR). **B**: Volcano plot of differentially expressed miRNAs. **C**: Dual luciferase gene reporter validated the interaction between lncRNA GTF3C1 and miR-542-3p. **Ci**: Potential binding site of miR-542-3p to the lncRNA GTF3C1 3' UTR and mutation site of the lncRNA GTF3C1-mut plasmid vector. **Cii**: Dual-luciferase gene reporter assay for relative luciferase activity after co-transfection of GTF3C1-wt and GTF3C1-mut plasmid vectors with miR-542-3p mimics or negative control in HEK293T cells (n = 3 per group). **D**: qRT‒PCR validated the differential expression of miR-542-3p in TG tissue of Ctrl, DM, Ad-NC treated diabetic mice (DM + Ad-NC), and Ad-GTF3C1 treated diabetic mice (DM + Ad-GTF3C1) (n = 3 per group) on day 5 after subconjunctival injection. **E**: Western blot analysis showing autophagy proteins expression in TG tissue of each group (n = 3 per group). **Ei**: Western blot bands of LC3B and P62 proteins. Quantified intensities of Western blot bands for LC3B (**Eii**) and P62 (**Eiii**) compared with β-actin. **F**: Immunofluorescence analysis showing LC3B protein expression in TG tissue of each group (n = 3 per group). **G**: Immunofluorescence analysis showing P62 protein expression in TG tissue of each group (n = 3 per group). Scale bar (including enlarged image): 50 μm. ns, not significant; *P < 0.05; **P < 0.01; ***P < 0.001; ****P < 0.0001

To explore the molecular mechanisms underlying the regulation of autophagy by the lncRNA GTF3C1, we first separated the nucleus and cytoplasm to determine the subcellular localization of the lncRNA GTF3C1. The results showed that more than 70% of the lncRNA GTF3C1 was expressed in the cytoplasm (Fig. 4A). lncRNAs distributed in the cytoplasm usually act as "molecular sponges" of miRNA. On the basis of previous miRNA sequencing results (GSE245193) in the TG tissue of diabetic mice, we manually identified two possible binding sites for the lncRNA GTF3C1 and miR-542-3p (Fig. 4B, Ci). To investigate the regulatory interaction between lncRNA GTF3C1 and miR-542-3p, we constructed a wild-type plasmid (GTF3C1-wt) and a mutant plasmid (GTF3C1-mut) with mutations in both binding sites (the two binding sites were spliced). The plasmids were co-transfected into human embryonic kidney 293T (HEK293T) cells with miR-542-3p mimics. The dual-luciferase reporter assay results demonstrated that miR-542-3p inhibited the luciferase activity of the wild-type lncRNA GTF3C1 reporter gene but had no significant effect on the luciferase activity of the mutant lncRNA GTF3C1 reporter gene (Fig. 4Cii). In addition, we subconjunctivally injected Ad-GTF3C1 into diabetic mice, and the expression of miR-542-3p in TG tissue was significantly lower than that in untreated diabetic mice (Fig. 4D). These results confirmed the targeting and binding relationships between lncRNA GTF3C1 and miR-542-3p.

Next, we used the agomir miR-542-3p to design a rescue experiment to verify whether the lncRNA GTF3C1 modulates autophagy in diabetic TG tissue through miR-542-3p. Following subconjunctival administration of Ad-GTF3C1 in diabetic mice, subsequent injection of the miR-542-3p agomir increased miR-542-3p expression levels. At the level of autophagy proteins, the miR-542-3p agomir counteracted the upregulation of LC3B and downregulation of P62 by Ad-GTF3C1 (Fig. 4E–G), suggesting that miR-542-3p reversed the agonistic effect of lncRNA GTF3C1 on autophagy in diabetic TG tissue. The above evidence indicated that lncRNA GTF3C1 targeted miR-542-3p to enhance the autophagy level in diabetic TG tissue.

### GABARAP and PTEN are potential targets of autophagy regulated by miR-542-3p in diabetic TG tissue

**Fig. 5:**
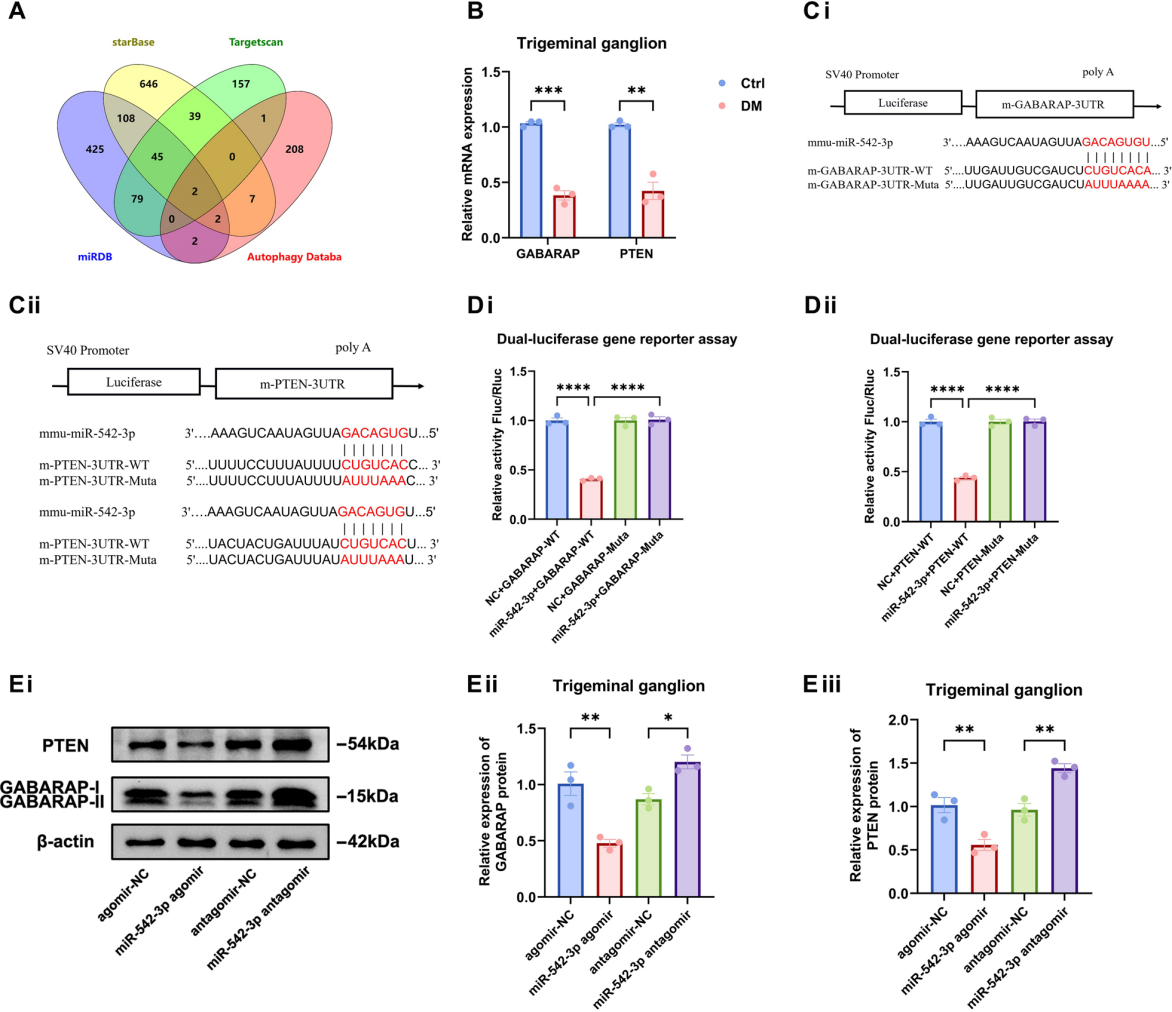
GABARAP and PTEN are potential targets of autophagy regulated by miR-542-3p in diabetic trigeminal ganglion (TG) tissue. **A**: The Venn diagram showed potential autophagy-related target genes of miR-542-3p were predicted in the Targetscan, StarBase, miRDB databases. **B**: Quantitative real-time polymerase chain reaction (qRT-PCR) validated the differential expression of GABARAP and PTEN in TG tissue of control (Ctrl) and diabetic (DM) mice (n = 3 per group). **C**: Potential binding site of miR-542-3p to PTEN and GABARAP (n = 3 per group). **Ci**: Potential binding site of miR-542-3p to the GABARAP 3' UTR and mutation site of the GABARAP-mut plasmid vector. **Cii**: Potential binding site of miR-542-3p to the GABARAP 3' UTR and mutation site of the GABARAP-mut plasmid vector. **D**: Dual-luciferase gene reporter assay for relative luciferase activity after co-transfection of plasmid vectors with miR-542-3p mimics or negative control in HEK293T cells (n = 3 per group). **Di**: Relative luciferase activity after co-transfection of GABARAP-WT and GABARAP-Muta plasmid vectors with miR-542-3p mimics or negative control. **Dii**: Relative luciferase activity after co-transfection of PTEN-WT and PTEN-Muta plasmid vectors with miR-542-3p mimics or negative control. **E**: Western blot analysis showing GABARAP and PTEN protein expression in TG tissue after subconjunctival injection with miR-542-3p agomir, agomir-NC, miR-542-3p antagomir, and antagomir-NC (n = 3 per group). **Ei**: Western blot bands of GABARAP and PTEN proteins. **Eii**: Quantified intensities of Western blot bands for GABARAP compared with β-actin. **Eii**: Quantified intensities of Western blot bands for PTEN compared with β-actin. *P < 0.05; **P < 0.01; ***P < 0.001; ****P < 0.0001

The function of miRNA is usually to induce the formation of RNA-induced silencing complexes (RISCs) to block target gene expression. To identify specific downstream genes regulated by miR-542-3p in autophagy, we used the TargetScan, StarBase, and miRDB databases to screen two autophagy-related target genes, PTEN and GABARAP (Fig. 5A), both of which had high binding scores. Moreover, qRT-PCR confirmed that the expression of both PTEN and GABARAP were significantly reduced in diabetic TG tissue. GABARAP, a member of the ATG8 protein family, is involved in critical cellular processes, including autophagy initiation, phagosome formation, and vesicle maturation [23]. PTEN exerts its regulatory function by negatively modulating the phosphatidylinositol 3-kinases/protein kinase B (PI3K/AKT) pathway, thereby influencing the downstream activity of mTORC1, which affects the initiation of autophagy. Through TargetScan, we obtained potential binding sites of miR-542-3p that are phylogenetically conserved in the 3' UTRs of GABARAP and PTEN and constructed wild-type and mutant plasmids of GABARAP and PTEN (Fig. 5C). The dual-luciferase reporter assay demonstrated a significant reduction in luciferase activity following the co-transfection of miR-542-3p mimics with wild-type GABARAP and PTEN plasmids. On the other hand, after the co-transfection of GABARAP and PTEN mutant plasmids and miR-542-3p mimics, luciferase activity did not change significantly (Fig. 5D). Next, we subconjunctivally injected the miR-542-3p agomir and miR-542-3p antagomir into diabetic mice to upregulate and silence miR-542-3p expression, respectively. The results indicate that the miR-542-3p agomir reduced the expression of the GABARAP and PTEN proteins in the TG tissue. The miR-542-3p antagomir upregulated the expression of GABARAP and PTEN proteins (Fig. 5D). Taken together, these demonstrate that miR-542-3p specifically interacts with GABARAP and PTEN. Hence, GABARAP and PTEN are potential targets for miR-542-3p to regulate autophagy in diabetic TG tissue.

### Co-silencing of GABARAP and PTEN abolishes lncRNA GTF3C1-mediated autophagy enhancement in diabetic TG tissue

**Fig. 6:**
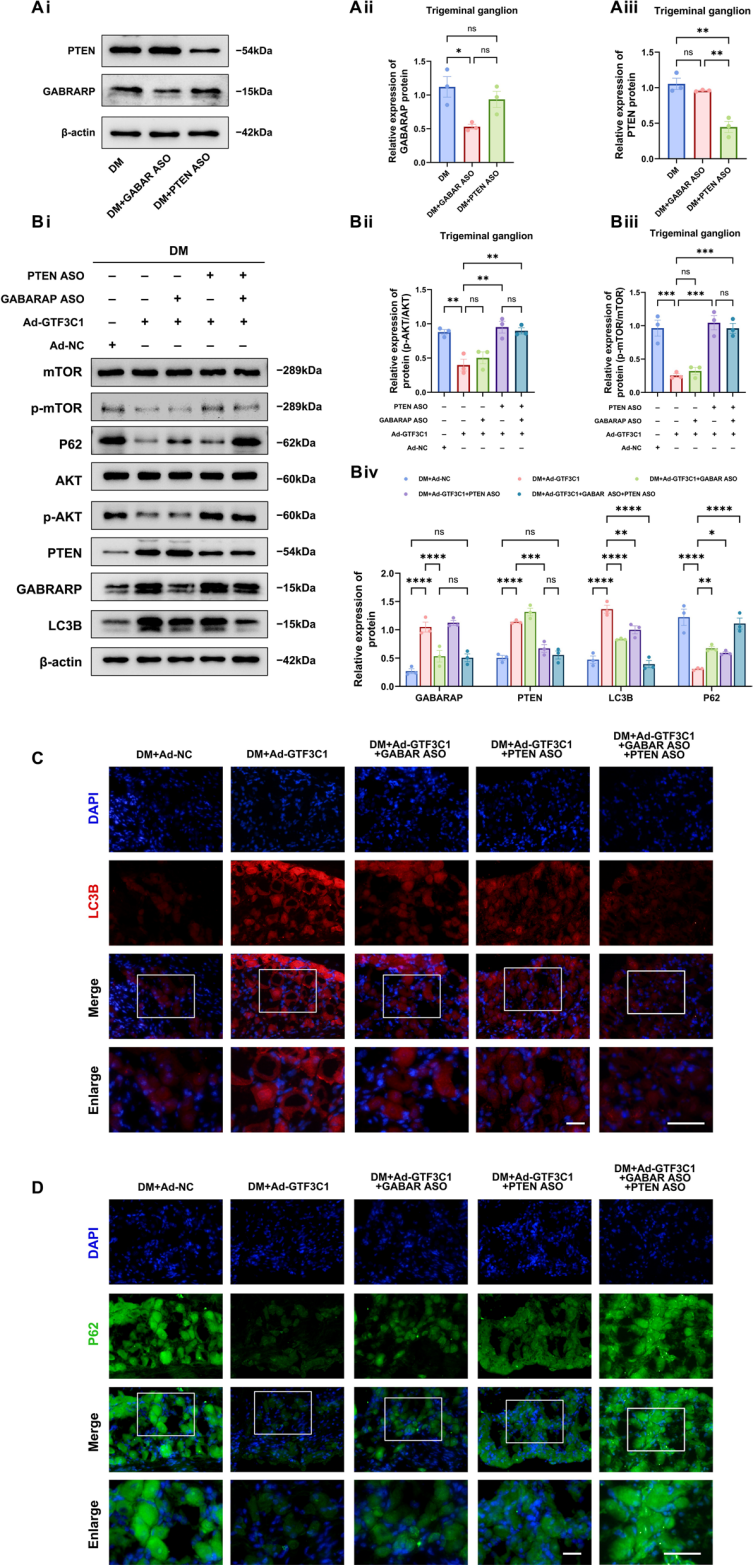
Co-silencing of GABARAP and PTEN abolishes lncRNA GTF3C1-mediated autophagy enhancement in diabetic trigeminal ganglion (TG) tissue. **A**: Western blot analysis detected the expression of GABARAP and PTEN proteins in TG tissue of diabetic mice (DM), diabetic mice injected with GABARAP ASO (DM + GABAR ASO), and diabetic mice injected with PTEN ASO (n = 3 per group). **Ai**: Western blot bands of GABARAP and PTEN proteins. **Aii**: Quantified intensities of Western blot bands for GABARAP and PTEN compared with β-actin. **B**: Western blot analysis detected the expression of GABARAP and PTEN proteins as well as autophagy proteins and PI3K/AKT pathway protein in diabetic TG tissues with PTEN or GABARAP knockdown after overexpression of LncRNA GTF3C1 (n = 3 per group). **Bi**: Western blot bands of proteins. **Bii**: Quantified intensities of Western blot bands for p-AKT compared with AKT. **Biii**: Quantified intensities of Western blot bands for p-mTOR compared with mTOR. **Biv**: Quantified intensities of Western blot bands for GABARAP, PTEN, LC3B, and P62 compared with β-actin. **C**: Immunofluorescence analysis showed LC3B protein expression in diabetic TG tissues with PTEN or GABARAP knockdown after overexpression of LncRNA GTF3C1 (n = 3 per group). **D**: Immunofluorescence analysis showed P62 protein expression in diabetic TG tissues with PTEN or GABARAP knockdown after overexpression of LncRNA GTF3C1 (n = 3 per group). Scale bar (including enlarged image): 50 μm. ns, not significant; *P < 0.05; **P < 0.01; ***P < 0.001; ****P < 0.0001

To explore whether the lncRNA GTF3C1 regulates autophagy in diabetic TG tissue through genes downstream of miR-542-3p, i.e., GABARAP and PTEN, we constructed antisense oligonucleotides (ASO) targeting GABARAP and PTEN. The Western blot confirmed that the subconjunctival injection of the GABARAP ASO successfully suppressed GABARAP protein in the TG tissue. Similarly, the PTEN ASO successfully knocked down the expression of the PTEN protein (Fig. 6A). Next, in addition to the subconjunctival injection of Ad-GTF3C1 into DM mice, we separately injected the PTEN ASO and GABARAP ASO to silence the expression of PTEN and GABARAP. Protein analyses revealed that the GABARAP ASO partially reversed the upregulated expression of the autophagy protein LC3B and the downregulated expression of P62 by the lncRNA GTF3C1 (Fig. 6B–D). The PTEN ASO suppressed autophagy by stimulating thePI3K/AKT signaling pathway, leading to elevated phosphorylated AKT and mTOR levels, which partially counteracted Ad-GTF3C1’s influence on autophagy proteins LC3B and P62 (Fig. 6B–D). In contrast, the simultaneous silencing of PTEN and GABARAP expression saw a near complete reversal of the regulatory effect Ad-GTF3C1 has on autophagy proteins LC3B and P62 (Fig. 6B–D). These results indicated that silencing GABARAP and PTEN reversed the enhancing effect of lncRNA GTF3C1 overexpression on the TG tissue in diabetic mice and that lncRNA GTF3C1 regulated the autophagy level in diabetic TG tissue largely through GABARAP and PTEN.

### The lncRNA GTF3C1 promotes nerve regeneration and epithelial repair in diabetic mice through PTEN and GABARAP

**Fig. 7:**
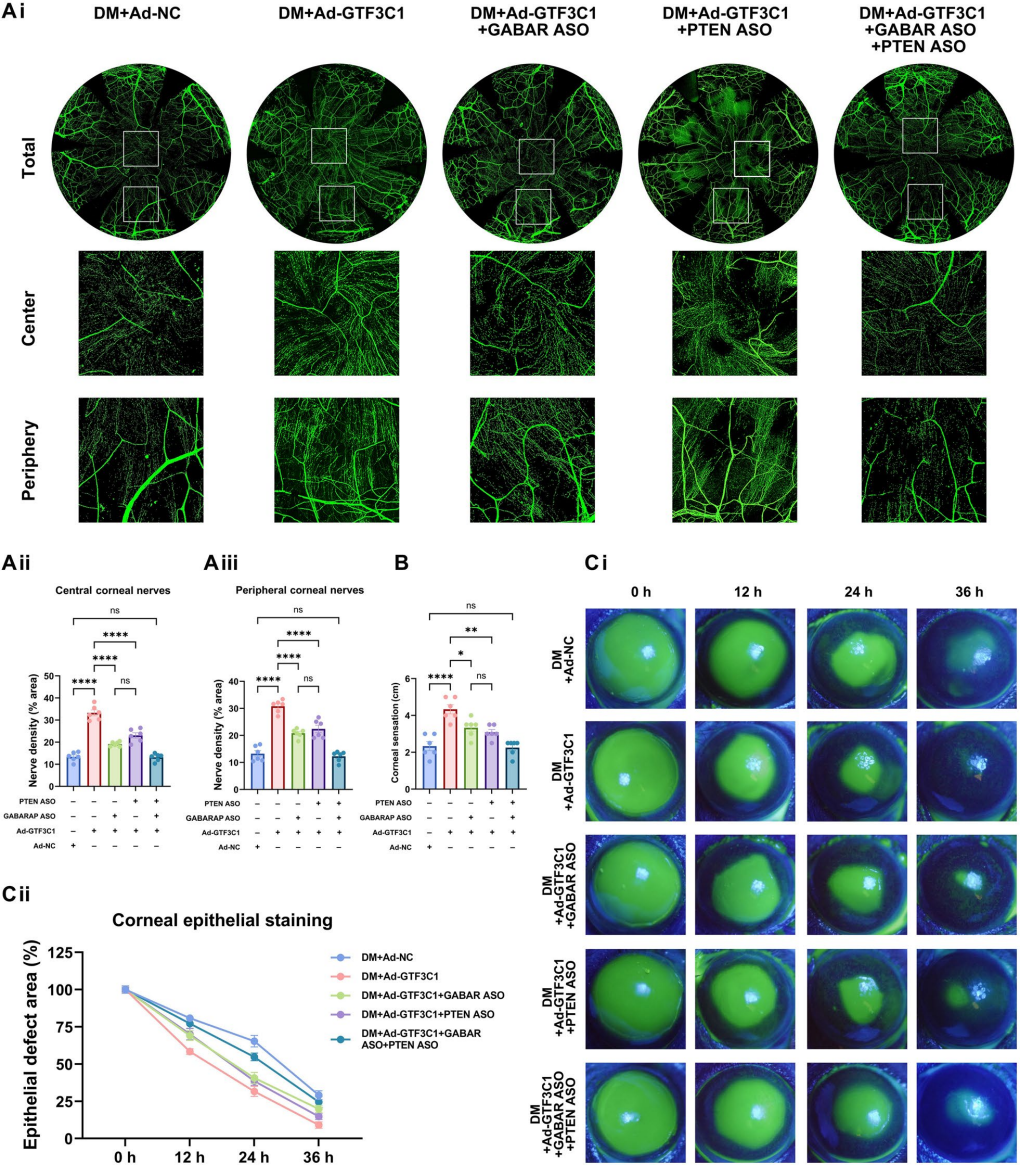
The lncRNA GTF3C1 promotes nerve regeneration and epithelial repair in diabetic mice (DM) through PTEN and GABARAP. **A** Corneal nerves whole-mount staining on day 5 of diabetic mice with PTEN or GABARAP knockdown after overexpression of LncRNA GTF3C1 (n = 6 per group). **Ai** Fluorescent images of corneal nerve staining. **Aii** The central corneal nerve density. **Aiii** The peripheral corneal nerve density. **B** Corneal sensation of each group on day 5 after debridement (n = 6 per group). **C** Corneas stained with fluorescein sodium of each group at 0, 12, 24, and 36 h after debridement (n = 6 per group). **Ci** Fluorescein-stained images of corneas. **Cii** Percentage of epithelial defect area. ns, not significant; *P < 0.05; **P < 0.01; ****P < 0.0001

To assess whether PTEN and GABARAP are involved in promoting the repair of diabetic corneal damage through lncRNA GTF3C1, we subconjunctivally injected Ad-GTF3C1 to overexpress the lncRNA GTF3C1 and then subconjunctivally injected the GABARAP ASO or the PTEN ASO to inhibit GABARAP or PTEN, respectively. We also simultaneously injected the GABARAP ASO and the PTEN ASO to jointly inhibit the expression of these two proteins. After the establishment of the corneal epithelial injury model, we found that compared with those in the Ad-NC-injection group, the corneal nerve density and corneal sensitivity were significantly higher in the Ad-GTF3C1-injection diabetic group on day 5 after injury, and the corneal epithelial healing rate was also significantly higher (Fig. 7A–C). GABARAP ASO treatment attenuated the pro-regenerative effects of lncRNA GTF3C1 on both corneal epithelial wound closure and nerve regeneration, while simultaneously restoring corneal sensitivity after diabetic corneal trauma (Fig. 7A–C). Similarly, the inhibition of PTEN expression alone partially antagonized the positive effects of lncRNA GTF3C1 in promoting diabetic corneal wound healing and nerve regeneration as well as improving corneal sensitivity (Fig. 7A–C). Notably, co-inhibition of GABARAP and PTEN signaling pathways almost completely abrogated the therapeutic benefits of lncRNA GTF3C1 in diabetic corneal repair, eliminating its positive effects on both epithelial restitution and sensory nerve regeneration. On day 5 of subconjunctival injection, there was no significant difference compared with the diabetic mice in the NC group in terms of corneal central and peripheral densities as well as corneal sensitivity (Fig. 7A–C). In summary, the lncRNA GTF3C1 enhanced autophagy and promoted corneal epithelial repair and nerve regeneration in diabetic mice through GABARAP and PTEN.

## Discussion

**Fig. 8:**
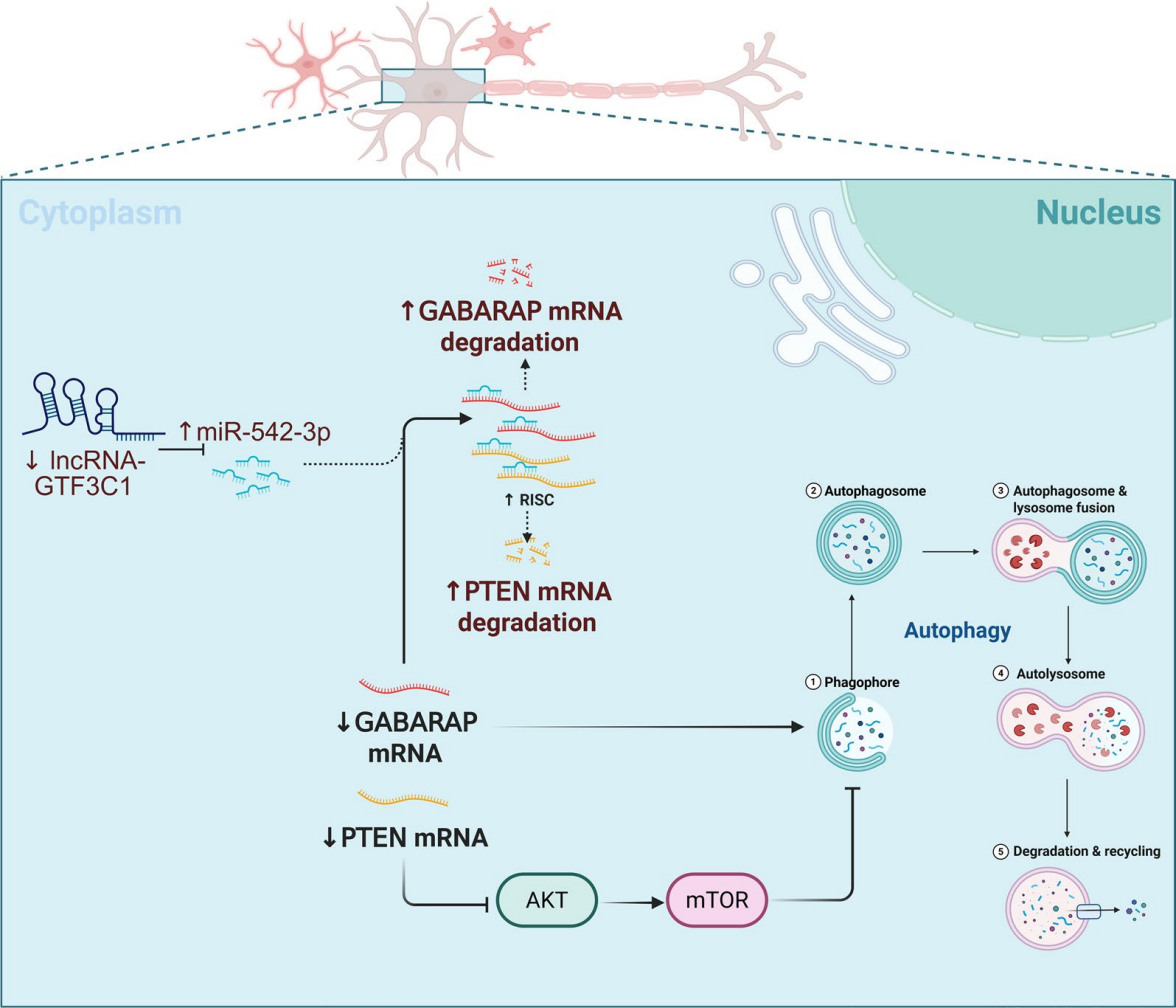
Ways by which lncRNA GTF3C1 regulates GABARAP and PTEN to activate autophagy in diabetic corneal nerve. GABARAP, GABA type A receptor-associated protein; mRNA, messenger RNA; lncRNA, long non-coding RNA; GTF3C1, general transcription factor IIIC subunit 1; PTEN, phosphatase and tensin homolog

As global diabetes prevalence escalates, the need for early intervention and improved DK therapies becomes increasingly critical. Corneal neuropathy – manifesting as impaired sensory function, trophic deficiency, and metabolic dysregulation – constitutes a key pathogenic driver of DK progression. Autophagy is a crucial biological process for the body's adaptive response as well as cellular renewal and repair, playing a key role in the healing of diabetic wounds [21, 22]. In this study, we demonstrate for the first time that the lncRNA GTF3C1 is involved in the regulation of autophagy in DCN. We report that the lncRNA GTF3C1 competitively binds to miR-542-3p, inhibits the silencing effect of miR-542-3p on its downstream genes GABARAP and PTEN, and thus enhance autophagy and promoting diabetic cornea wound healing (Fig. 8).

Previous studies have indicated the potential of autophagy regulation as a therapeutic strategy for diabetic corneal wound healing [13]. This is the first study to propose a novel effect regarding lncRNA transcripts in regulating autophagy within diabetic TG tissue. We identified downregulated lncRNA GTF3C1 in diabetic trigeminal ganglia. Its overexpression enhanced diabetic corneal repair while elevating autophagic flux, whereas autophagy inhibition abolished these therapeutic effects, demonstrating lncRNA GTF3C1 acts through autophagy induction.

Cytoplasmic lncRNAs typically function as miRNA sponges, competitively inhibiting target gene silencing. We observed cytoplasmic enrichment of lncRNA GTF3C1 and identified miR-542-3p as its binding target. miR-542-3p has been confirmed to participate in diabetic cornea wound healing [13]. Its documented roles in neuroblastoma invasion and microglial pyroptosis further suggest neurological disease relevance [23, 24]. Through bioinformatics analysis, we predicted that GABARAP and PTEN are potential target genes through which miR-542-3p regulates autophagy. GABARAP is a crucial member of the ATG8-family of proteins. The C-terminal residues of ATG8-family of proteins undergo ubiquitin-like conjugation with the lipid phosphatidylethanolamine on the phagophore membrane, which is necessary for autophagy membrane elongation together with autophagosome maturation and closure processes [25]. PTEN is a lipid phosphatase that inhibits the PI3K/AKT signaling pathway activation and mobilizes the downstream molecule mTORC1, which inhibits the initiation of autophagy through phosphorylating ULK in the autophagy pathway [26]. We confirmed that the dual silencing of GABARAP and PTEN abolished the pro-autophagic and wound-healing effects of lncRNA GTF3C1, confirming its role as a miR-542-3p sponge that enhances diabetic corneal repair via autophagy regulation.

While this study identifies the GTF3C1/miR-542-3p axis as a key autophagy regulator in DCN, the inherent complexity of lncRNA/miRNA networks must be acknowledged [27, 28]. PTEN functions as a pleiotropic regulator that interfaces with both autophagic and apoptotic/inflammatory pathways [29, 30]. Although our current findings emphasize autophagy-mediated nerve repair, the GTF3C1/miR-542-3p axis potentially exerts concurrent effects on corneal apoptosis and inflammation under diabetic conditions. Systematic exploration of this crosstalk remains essential to fully evaluate its therapeutic potential in DK.

## Conclusions

In conclusion, our study delineates the critical role of the lncRNA GTF3C1/miR-542-3p/autophagy axis in diabetic corneal wound healing. These findings suggest two key clinical implications. First, lncRNA GTF3C1 expression and autophagy flux monitoring could enhance diagnostic evaluation for diabetic patients with ocular surface complications, potentially enabling earlier detection of DK. Second, therapeutic targeting of this pathway through non-invasive approaches (e.g., molecularly engineered topical formulations) may represent a promising treatment strategy for diabetic corneal disease. Several limitations should be acknowledged in this study. The high mortality rate in diabetic animal models constrained the achievable sample size, potentially impacting statistical power. To address this, future investigations will employ multi-center collaborative studies to increase cohort sizes, and complementary organoid-based platforms to reduce animal use while maintaining physiological relevance. These approaches will strengthen the translational foundation for clinical application of these findings.

## Supplementary Information

**Additional file 1.**

## Data Availability

All data are included in the manuscript and supplementary materials. The resources during the current study are available from the corresponding author upon reasonable request.

## Abbreviations

3-MA: 3-Methyladenine
Ad: Adenovirus
ASO: Antisense oligonucleotide
ATG: Autophagy related gene
DCN: Diabetic corneal neuropathy
DELncRNA: Differentially expressed LncRNA
DK: Diabetic keratopathy
GABARAP: GABA type A receptor-associated protein
GTF3C1: General transcription factor IIIC subunit 1
HEK: Human embryonic kidney
LC3B: Light chain 3 beta
LncRNA: Long non-coding RNA
miRNA: MicroRNA
P62: Sequestosome-1
PI3K-AKT: Phosphatidylinositol 3-kinases/protein kinase B
PTEN: Phosphatase and tensin homolog
qRT-PCR: Quantitative real-time polymerase chain reaction
RISC: RNA-induced silencing complex
STZ: Streptozotocin
TG: Trigeminal ganglion
