# Supplementary material for: LncRNA GTF3C1 promotes diabetic corneal wound healing by regulating GABARAP and PTEN to augment autophagy
Source: Eye Vis (Lond). 2025 Aug 11;12:33. doi: 10.1186/s40662-025-00448-y (PMC12337447; doi:10.1186/s40662-025-00448-y)

**Supplemental Table S1.** Solutions used for subconjunctival injections.

| **Injection solutions** | **Concentration (μmol/L)** | **Manufacturer** |
| --- | --- | --- |
| Adenovirus-GTF3C1 | 20 | Hanbio, Shanghai, China |
| Adenovirus-NC | 20 | Hanbio, Shanghai, China |
| miR-542-3p agomir | 200 | Ribobio, Guangzhou, China |
| miR-542-3p antagomir | 200 | Ribobio, Guangzhou, China |
| agomir-NC | 200 | Ribobio, Guangzhou, China |
| antagomir-NC | 200 | Ribobio, Guangzhou, China |
| PTEN ASO | 200 | Ribobio, Guangzhou, China |
| GABARAP ASO | 200 | Ribobio, Guangzhou, China |

**Supplemental Table S2.** Primary antibodies for Western blot and Immunofluorescence.

| **Antibody** | **Catalog number** | **Manufacturer** | **Dilution used** |
| --- | --- | --- | --- |
| anti-p62 | P0067 | Sigma-Aldrich, USA | WB 1/1000  IF 1/200 |
| anti-LC3B | ab192890 | Abcam, USA | WB 1/1000  IF 1/200 |
| anti-GABARAP | ab109364 | Abcam, USA | WB 1/2000 |
| anti-β-actin | ab8226 | Abcam, USA | WB 1/1000 |
| anti-PTEN | 9559 | CST, USA | WB 1/1000 |
| anti-AKT | 9272 | CST, USA | WB 1/1000 |
| anti-pSer472 AKT | 4060 | CST, USA | WB 1/2000 |
| anti-mTOR | 2983 | CST, USA | WB 1/1000 |
| anti-pSer2448-mTOR | 2971 | CST, USA | WB 1/1000 |

**Supplemental Table S3.** Primer sequences for conventional quantitative real-time polymerase chain reaction (qRT-PCR).

| **Gene** | **Forward primer (5′-3′)** | **Reverse primer (5′-3′)** |
| --- | --- | --- |
| GABARAP | ACAAAAAGAAATACCTGGTGCC | GGTGGAATGACATTGTTGACAA |
| PTEN | GGAAAGGGACGGACTGGTGTAATG | CGCCTCTGACTGGGAATTGTGAC |
| β-actin | GTACCACCATGTACCCAGGC | AACGCAGCTCAGTAACAGTCC |
| U6 | CCTGCTTCGGCAGCACA | AACGCTTCACGAATTTGCGT |
| miR-542-3p | GCCGTGTGACAGATTGATAACTGA | AACGCTTCACGAATTTGCGT |
| GTF3C1* | GAATGCTGTGTCGGCTCCTTC | TGTCGGCTCAAGTCACTCTCC |
| STK16* | TGAGCAGCAAGACCAGGAAGAAG | ATGAGGCGAAGGATGTTGGGATG |
| SEMA5A* | TCACACTGCTTGTCTACACCTACTG | GTGGTTAGTTATGCTGCTGTTGAGG |
| RWDD4A* | GTGGCTCAGTCAGTAAAGGTTTGC | GAACCAGGTGCTCAGTGACTCAG |
| RSAD1* | GTCTACCTGCCTGCCGATTCG | CGTCGCTGGCTGAGTGAGTC |
| MRPL58* | TGTCTGCTTAGGTGGTTGGCTATC | CACGGGCTCCTCAATCCAGTC |
| KAT2A* | TGAGCGAGTTGTGCCGTAGC | TTGGTGTCTGTGTCCTCTTCCTTG |
| FKBP1A* | TGATTCCTCTCGGGACAGAAACAAG | CGGTGGCTCCATAGGCATAGTC |
| DOPEY2* | ACCACTAATCTCCCGCTTGCTATAC | ACAGCTCCAGCACTTCCTTCTTC |

* LncRNA transcripts were denoted by their gene names. Primers for all lncRNA transcripts were designed based on spliced cDNA sequences.

**Supplemental Table S4**. Identified up-regulated lncRNAs from RNA-seq.

| **Trans_Name** | **log_2_FC** | **Fold_Change** | ***P* value** |
| --- | --- | --- | --- |
| Stk16-207 | 3.246075295 | 9.487811158 | 0.000001327 |
| Fkbp1a-203 | 2.5941129 | 6.03817638 | 0.000271083 |
| Kat2a-204 | 2.111705305 | 4.322018671 | 0.000452163 |
| Rwdd4a-205 | 1.972051518 | 3.923256112 | 0.000095118 |
| Mrpl58-210 | 1.923111931 | 3.792402082 | 0.027230026 |
| Arfip2-205 | 1.897781354 | 3.726396924 | 0.007101699 |
| Orc5-202 | 1.882799917 | 3.687900972 | 0.010114098 |
| 4930453N24Rik-202 | 1.721406196 | 3.297576654 | 0.000000008 |
| Slc9a8-206 | 1.579389186 | 2.988432974 | 0.024835409 |
| Trrap-206 | 1.489017986 | 2.806978445 | 0.015321160 |
| Idh3g-203 | 1.451598794 | 2.735109882 | 0.013170343 |
| Psmc3-209 | 1.372434738 | 2.589071376 | 0.031114809 |
| Chchd10-202 | 1.331175711 | 2.516076368 | 0.006074232 |
| Nalcn-204 | 1.301545331 | 2.4649277 | 0.006645002 |
| Msto1-203 | 1.29249387 | 2.449511169 | 0.001064604 |
| Vwa5b2-201 | 1.278541352 | 2.425935767 | 0.001923808 |
| Kat2a-203 | 1.27195817 | 2.414891163 | 0.001925550 |
| F630028O10Rik-202 | 1.226651223 | 2.340231455 | 0.008368877 |
| Mrpl21-203 | 1.200219106 | 2.297745648 | 0.000314945 |
| Paxbp1-204 | 1.173532273 | 2.255632873 | 0.002860405 |

**Supplemental Table S5.** Identified down-regulated lncRNAs from RNA-seq.

| Trans_Name | log_2_FC | Fold_Change | *P*_value |
| --- | --- | --- | --- |
| Sema5a-203 | −3.395128972 | 0.095052674 | 0.000128733 |
| Gtf3c1-205 | −2.110268626 | 0.231603887 | 0.035691794 |
| Dhx32-207 | −1.730567135 | 0.301333477 | 0.013883983 |
| Rsad1-202 | −1.723701275 | 0.302770956 | 1.78907E-05 |
| Dopey2-208 | −1.652404112 | 0.318109616 | 4.44235E-05 |
| Atl2-204 | −1.385060183 | 0.382873527 | 0.00553725 |
| Nudt19-203 | −1.36225466 | 0.388973922 | 0.003368779 |
| Cars2-211 | −1.273847634 | 0.413555358 | 0.003279557 |
| Tecr-204 | −1.234323582 | 0.425041737 | 0.009628489 |
| Tnks-203 | −1.13686562 | 0.454746481 | 0.034181172 |
| Ankrd42-206 | −1.120335061 | 0.459986983 | 0.029899319 |
| Plk3-202 | −1.068343454 | 0.476866237 | 0.025805701 |
| Eif4g3-209 | −1.02301475 | 0.49208698 | 0.036777423 |
| 9930104L06Rik-201 | −1.007621475 | 0.497365563 | 0.023395342 |
| Commd8-204 | −0.987031645 | 0.504514751 | 0.00912481 |
| Acad9-204 | −0.983645198 | 0.505700392 | 0.024893934 |
| Ehf-206 | −0.959413516 | 0.51426593 | 0.040812646 |
| Casp12-202 | −0.955723626 | 0.515582919 | 0.030900377 |
| Jkamp-206 | −0.924501685 | 0.526862468 | 0.027484589 |
| Eri3-206 | −0.896136387 | 0.537323788 | 0.042689962 |

**Supplemental Figure S1. Ocular surface toxicity test of Adenovirus by subconjunctival injection.** **A**: Intraocular pressure of diabetic mice injected with PBS (DM+PBS), diabetic mice injected with Ad-NC (DM+Ad-NC) and diabetic mice injected with Ad-GTF3C1 (DM+Ad-GTF3C1) (n = 6 per group). Corneal transparency (**B**), corneal neovascularization (**C**), and corneal epithelium (**D**) in each group on day 7 (n = 6 per group). **E**: Hematoxylin and eosin (HE) staining observation of corneal thickness in each group on day 7 (n = 6 per group). **Ei**: HE staining of the corneal section. **Eii**: Measurement of corneal thickness. ns, *P* > 0.05.


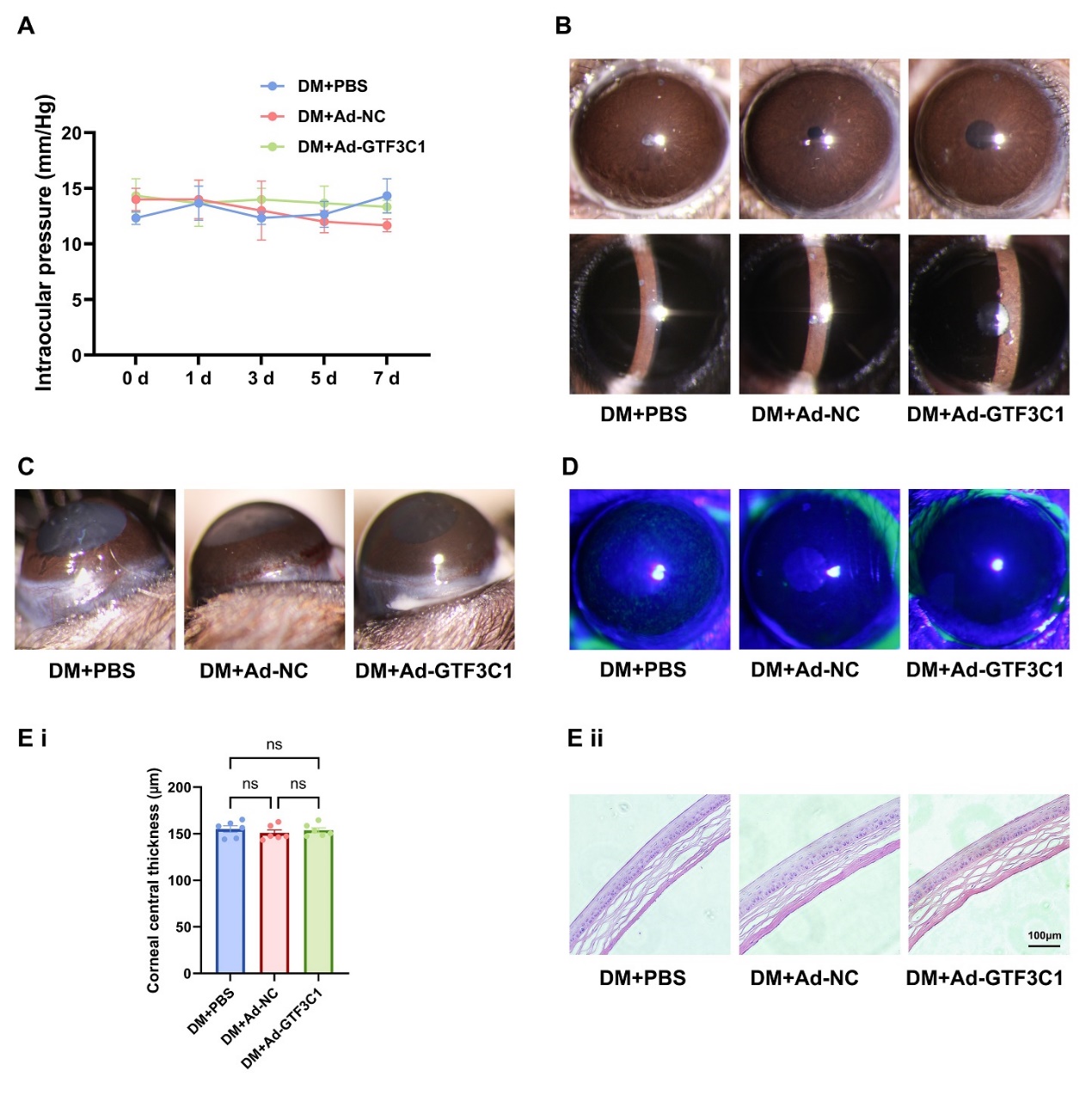

Supplement: Supplementary file 1 — Additional file 1. [file 40662_2025_448_MOESM1_ESM.docx]
